# Multilevel Resistive Switching Memory Based on a CH_3_NH_3_PbI _3−*x*_Cl_*x*_ Film with Potassium Chloride Additives

**DOI:** 10.1186/s11671-020-03356-3

**Published:** 2020-06-05

**Authors:** Fengzhen Lv, Kang Ling, Tingting Zhong, Fuchi Liu, Xiaoguang Liang, Changming Zhu, Jun Liu, Wenjie Kong

**Affiliations:** College of Physics and Technology, Guangxi Normal University, Yucai Road, Guilin, 541000 China

**Keywords:** Tri-state resistive switching behavior, KCl-doped MAPIC films, Iodine vacancies, Trap-controlled SCLC conduction mechanism

## Abstract

High-quality CH_3_NH_3_PbI _3−*x*_Cl_*x*_ (MAPIC) films were prepared using potassium chloride (KCl) as an additive on indium tin oxide (ITO)-coated glass substrates using a simple one-step and low-temperature solution reaction. The Au/KCl-MAPIC/ITO/glass devices exhibited obvious multilevel resistive switching behavior, moderate endurance, and good retention performance. Electrical conduction analysis indicated that the resistive switching behavior of the KCl-doped MAPIC films was primarily attributed to the trap-controlled space-charge-limited current conduction that was caused by the iodine vacancies in the films. Moreover, the modulations of the barrier in the Au/KCl-MAPIC interface under bias voltages were thought to be responsible for the resistive switching in the carrier injection trapping/detrapping process.

## Introduction

As a result of the rapid development in the information storage industry, the high storage density is important for the memory technology. Along with the limit size (∼ 22 nm) of silicon-based memories is approaching, it is difficult to obviously enhance the storage density through further scaling down the device size. Thus, the multilevel storage is an effective alternative approach to enhance the storage density [1, 2]. Among various types of modern memories, resistive switching random access memory (ReRAM) has attracted remarkable attention owing to its simple cell architecture, fast programming speed, high storage density, and low power consumption [3–6]. The ability of multilevel resistive switching (RS) effect has been reported in various inorganic materials [7–10]. Although they possess excellent memory performance, the complex fabrication process and rigidity hinder their development for ReRAM. Most recently, organometal halide perovskites (OHPs) have attracted a great deal of attention in the ReRAM due to their high flexibility, tunable band gaps, and large absorption coefficients [11–15]. Additionally, OHPs have highly defect-tolerant, facile, and cost-effective solution-processed methods that can be adopted to fabricate the OHPs layers [16, 17]. However, OHP-based ReRAM suffers from poor endurance and retention performance. These drawbacks are related to the poor quality of OHP films [18, 19]. In the most recent studies, potassium halides have been proposed as additives to effectively reduce the grain boundaries and compensate for defects in OHPs, to improve optoelectronic properties of OHPs [19–21]. Nevertheless, the RS behavior in potassium halide-doped OHPs has not been extensively reported.

In this study, we prepared CH _3_*NH*_3_*PbI*_3−*x*_Cl_*x*_(MAPIC) films with the potassium chloride (KCl) additive on indium tin oxide (ITO)-coated glass substrates using a one-step low-temperature solution treatment. Distinct multilevel RS behavior was achieved by the Au/KCl-MAPIC/ITO/glass devices at different set voltages (*V*_SETs_). Subsequently, we analyzed the non-volatile RS effect in the Au/KCl-MAPIC/ITO/glass memory device. The electrical conductive behavior is primarily attributed to the trap-controlled space-charge-limited current (SCLC) conduction mechanism based on the variation of iodine vacancies in the KCl-MAPIC films. Moreover, the modulations of the barrier at the Au/KCl-MAPIC interface under bias voltages are thought to be responsible for the RS behavior.

## Methods

**Fig. 1:**
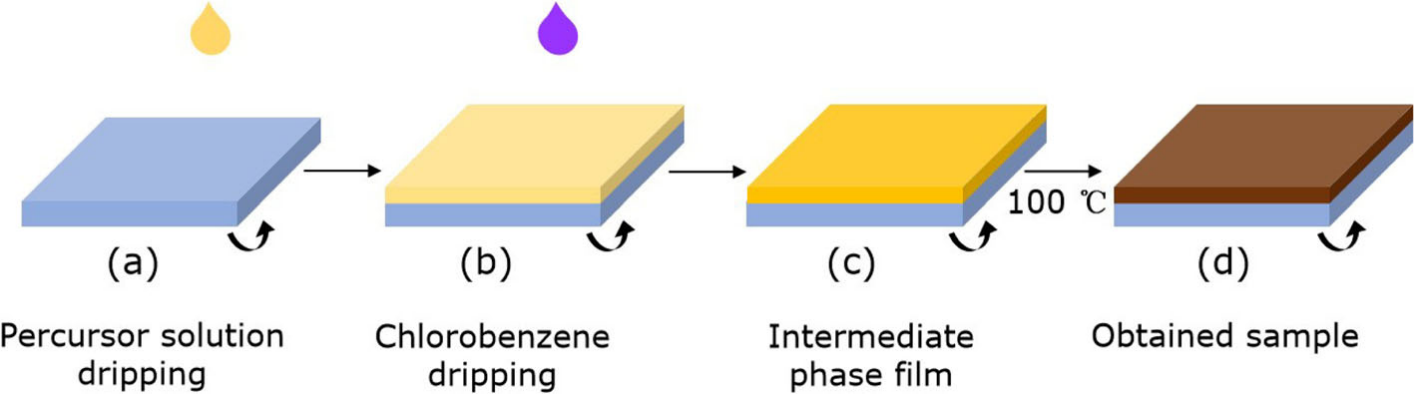
Solvent engineering procedure for preparing the KCl-doped MAPIC film on the ITO-coated glass substrate

Prior to growing the samples, the ITO/glass substrates (10 mm ×10 mm, Luoyang Guluo Glass Co., Ltd.) were cleaned sequentially in acetone, isopropyl alcohol, and deionized water and were dried under a nitrogen gas flow. The perovskite precursor solution was prepared by combining lead iodide (PbI_2_, 98%, 370 mg, Shanghai Aladdin Bio-Chem Technology Co., Ltd.), methylammonium iodide (MAI, 99.5%, 130 mg, Shanghai Macklin Biochemical Co., Ltd.), and methylammonium chloride (MACl, 98%, 20 mg, Shanghai Aladdin Bio-Chem Technology Co., Ltd.) with anhydrous *N,N*-dimethylformamide (DMF, >99.5%, 1 mL, Xilong Scientific Co., Ltd.). Subsequently, KCl (>99.5%, 7 mg, Tianjin Guangfu Technology Development Co., Ltd.) was added to the mixed solution. The yellowish precursor solution (0.8 M) was stirred more than 6 h in an argon-filled glove box. Then, the precursor solution was spin-coated on ITO/glass substrates at 3000 rpm for 30 s, as shown in Fig. 1a. After 6 s of spin coating, anhydrous chlorobenzene (100 *μ*L, Shanghai Aladdin Bio-Chem Technology Co., Ltd.) was dropped rapidly onto the surface of the intermediate phase film. The film immediately changed from pale yellow to nut-brown [Fig. 1b, c]. Finally, the sample was heated on a hot plate at 100 ^∘^C for 10 min, as shown in Fig. 1d.

## Characterization

The crystal structure of the MAPIC films was investigated by X-ray diffractometry (XRD; MiniFlex600, Rigaku, JPN). The chemical element analysis of the films was performed using X-ray photoelectron spectroscopy/ultraviolet photoelectron spectroscopy (XPS/UPS; ESCALAB250Xi, Thermo Fisher Scientific, USA) using Al K *α* radiation and a He I source with 21.22 eV. The surface morphology of the MAPIC films was examined using scanning electron microscopy (SEM; FEI Quanta 200). The electrical characterization of the KCl-MAPIC films was performed using a Keithley 2400 SourceMeter controlled by the LabVIEW program.

## Results and Discussion

**Fig. 2:**
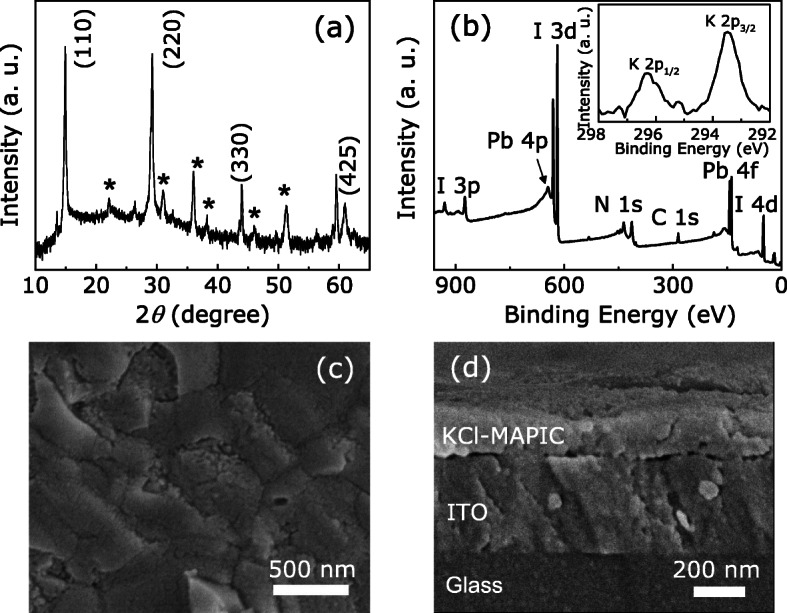
**a** XRD spectrum of the as-prepared KCl-doped MAPIC films on the ITO-coated glass substrate. ⋆ represents peaks of the ITO/glass substrate. **b** XPS wide spectrum of the perovskite films. The inset displays the core-level XPS spectrum of K. **c** The top-view and **d** the cross-section SEM images of KCl-MAPIC layers formed on the ITO/glass substrate

Figure 2a shows the XRD pattern of the KCl-doped MAPIC films. The (110), (220), and (330) sharp peaks are in accordance with the tetragonal phase of the crystallized perovsikte film [12, 22]. Figure 2b depicts the XPS wide scan spectrum of the KCl-MAPIC films. C, Pb, I, N, and K are obviously present in the films. However, the peak of Cl 2*p* core-level can not be clearly observed in the full spectrum. This finding is consistent with the results of previous reports, where a number of Cl atoms that involve in the form of gaseous CH_3_NH_3_Cl or other gaseous Cl-containing mixtures could easily escape in the annealing step, in order to drive the formation and crystallization of perovskite films [22, 23]. Although the XPS wide scan spectrum shows negligible signals of the Cl 2*p* core level, the narrow scan detects weak signals corresponding to the Cl 2*p*_3/2_ and Cl 2*p*_1/2_ peaks, as shown in Additional file 1: Fig. S1 (Supporting Information). It indicates that there are minute amounts of Cl in the final product of perovskite films. Figure 2c presents the top-view SEM image of the KCl-MAPIC films. It is found that the KCl-doped MAPIC films exhibit a high coverage and is dense. Compared with the porous surface of MAPIC films without the KCl additive (Additional file 1: Fig. S2), KCl as a kind of suitable additive is demonstrated that can enhance the quality of OHP films. It consists to previous reports, in which the alkali metal halides could chelate with Pb ^2+^ ions and enhance the crystal growth of lead-halide perovskite films [19, 24]. Figure 2d shows that the thickness of the dense KCl-MAPIC layer is ∼ 200 nm.

**Fig. 3:**
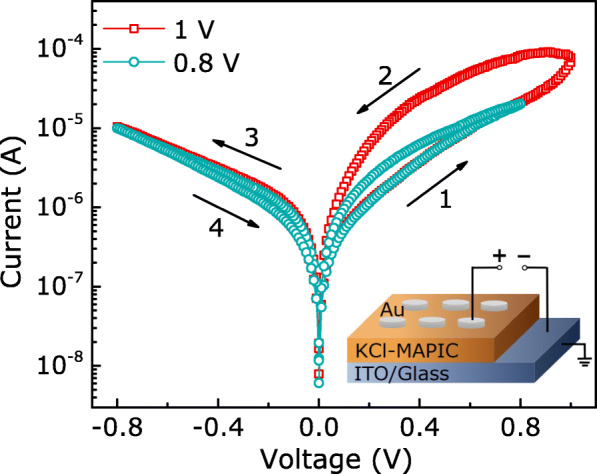
The semi-logarithmic plots of the *I*−*V* curves of Au/KCl-MAPIC/ITO/glass devices in voltage sweeping mode. The inset displays the schematic measurement. Au electrodes with diameters of 300 *μ*m were deposited on the surface of KCl-MAPIC films by magnetron sputtering

Figure 3 shows the current-voltage (*I*−*V*) characteristics by applying voltage loops to the Au/KCl-MAPIC/ITO/glass devices with periodic sweepings (0 V →0.8 V/1 V →0 V →-0.8 V →0 V). Initially, the device is in a high-resistance state (HRS), and then, the current increases gradually as the positive voltage increases. Subsequently, the memory device transitions from the HRS to different low-resistance states (LRSs) under the two *V*_SETs_ of 0.8 V and 1 V. The *I*−*V* characteristics indicate that the Au/KCl-MAPIC/ITO/glass devices have the multilevel storage potential.

In order to identify the RS performance of Au/KCl-MAPIC/ITO/glass devices, we measured the *I*−*V* curves of the devices based on MAPIC films without the KCl additive as references. As shown in Additional file 1: Fig. S3(a), a typical bipolar RS behavior is observed in MAPIC films prepared without the KCl doping, whereas the RS effect is weaker than in KCl-doped MAPIC films. As exhibited in Additional file 1: Fig. S3(b), the multilevel RS behavior is not been observed in the Au/MAPIC/ITO/glass devices under the *V*_SETs_ of 0.8 V and 1.0 V. The above results indicate that the KCl additive improves memory properties of the MAPIC-based devices. We surmise that the improvement is related with the enhancement of the films quality. The dense surface of KCl-doped MAPIC films avoided the top electrodes getting deposited in the pores and directly contacting with the bottom electrodes during the preparation process of the devices. So it is helpful to growing the uniform RS structures with the OHP layers [19, 25].

**Fig. 4:**
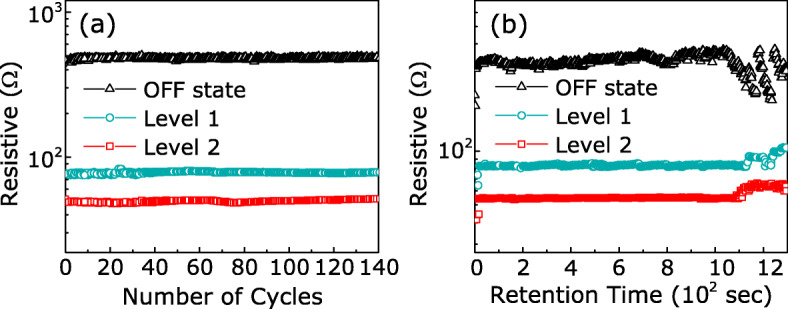
**a** Pulse cycles up to 140 times and **b** time up to about 1200 s for HRS and LRS measurements in the Au/KCl-MAPIC/ITO/glass device at room temperature

The retention and endurance stability determine the multilevel-storage reliability of the Au/KCl-MAPIC/ITO/glass devices and evaluate the potential application of the devices in the RRAM. Figure 4a displays the endurance cycle dependence of the resistance states in the Au/KCl-MAPIC/ITO/glass devices. Electric pulses of reset voltage (*V*_RESET_) and *V*_SETs_ were alternately applied to the devices (pulse width=0.4 s). After applying the *V*_RESET_ of –0.8 V, a high resistance state (HRS) was measured at a read voltage (*V*_r_=0.22 V), which was defined as the “OFF state.” After applying the *V*_SETs_ of 0.8 V and 1 V, two different low resistance states (LRSs) were measured at the *V*_r_, which were defined as “level 1” and “level 2,” respectively. Above different resistance states can be maintained for up to 140 cycles under electric pulses. Figure 4b displays the retention property of the Au/KCl-MAPIC/ITO/glass devices. After applying the *V*_RESET_, the device showed “OFF state” at the *V*_r_ and maintained “OFF state” after the *V*_RESET_ was removed. After applying *V*_SETs_, the device exhibited “level 1” and “level 2” at the *V*_r_; these two LRSs remained even though *V*_SETs_ were removed. Each resistance state is stable for over 1000 s without operation voltages. Therefore, the potential of the multilevel memory has been demonstrated in the Au/KCl-MAPIC/ITO devices.

**Fig. 5:**
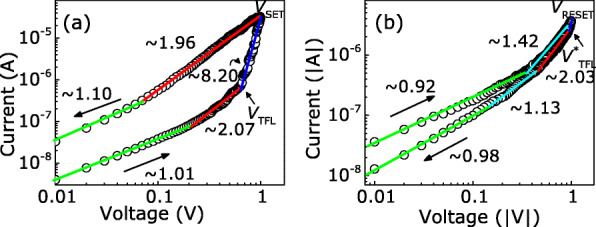
The fitted lines of log *I*-log *V* plots in the **a** positive and **b** negative voltage regions. The arrows indicate the sweeping direction

In order to investigate the mechanism of RS behaviors in the Au/MAPIC/ITO/glass devices, the relationship of log *I* versus log *V* was plotted. As shown in Fig. 5a, in the initial positive bias region from 0 to 0.2 V, the *I*−*V* relationship has a slope of ∼ 1.01, demonstrating that the conductive behavior follows Ohm’s law. With the positive bias increasing (0.2 V ∼ 0.6 V), the *I*−*V* relationship is *I*∝*V*^2^ and obeys the SCLC mechanism controlled by single shallow traps. When the forward bias reaches the trap-filled limit voltage (*V*_TFL_), the current increases sharply with the bias voltage sweeping and the slope is ∼ 8.20, and the *I*−*V* relationship obeys the exponential distributed trap-controlled SCL conduction. When the bias reaches *V*_SET_, the resistive state changes into the LRS. Even though the positive bias decreases, the resistance still maintains the LRS. As illustrated in Fig. 5b, when the bias sweeps reversely, the Au/KCl-MAPIC/ITO/glass device remains in the LRS, whereas the negative bias crosses *V*_RESET_ and reaches $V^{*}_{\text {TFL}}$; the current decreases as the voltage decreases and the relationship of *I*−*V* recovers *I*∝*V*^2^.

**Fig. 6:**
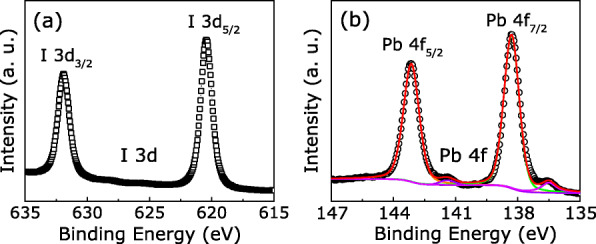
XPS spectra of **a** I 3*d* and **b** Pb 4*f*core levels of the KCl-doped MAPIC films

In OHP-based ReRAM, it is generally accepted that intrinsic point defects in OHP layers can be responsible for the RS behavior [26]. Thereinto, halide vacancies are readily formed in the OHP films during the lowest solution-based film-deposition process [27]. Among these vacancies, iodine vacancy ($\mathrm {V}_{\dot {\mathrm {I}}}$) possesses a high mobility due to the lowest activation energy of ∼ 0.58 eV [26, 28]. Thus, $\mathrm {V}_{\dot {\mathrm {I}}}$ is assumed to play a major role for the RS conductive behavior in the Au/KCl-MAPIC/ITO/glass devices [29]. Additionally, although the appropriate dosage of KCl additives can enhance the MAPIC film quality, potassium ion doping has been verified that could suppress hysteresis of current in OHP solar cells due to the compensation effect for the defect states at the surface or interface of OHP layers [19, 21, 30]. Thus, the origin of obvious multilevel RS characteristics is hardly attributed to potassium ions in our work. We obtained XPS measurements to verify the hypothesis and analyze the condition of the perovskite layer. Figure 6 illustrates the survey XPS spectra of I 3*d* and Pb 4*f*. The peaks located at 631.90 eV and 620.45 eV are consistent with I 3 *d*_3/2_ and I 3 *d*_5/2_, respectively. The peak positions shift slightly to higher binding energy, which indicates the generation of $\mathrm {V}_{\dot {\mathrm {I}}}$ by heat-driven deiodination [31, 32]. The XPS result in Fig. 6b shows the Pb 4 *f* core level spectrum. Two main peaks of Pb 4 *f*_5/2_ and Pb 4 *f*_7/2_ are observed at 143.18 eV and 138.21 eV, respectively. It is noteworthy that additional small peaks with lower binding energies (141.41 eV and 136.60 eV) with the signature of Pb^0^ were detected by XPS [33, 34]. These results indicate that $\mathrm {V}_{\dot {\mathrm {I}}}$ exists in the KCl-doped MAPIC layer.

**Fig. 7:**
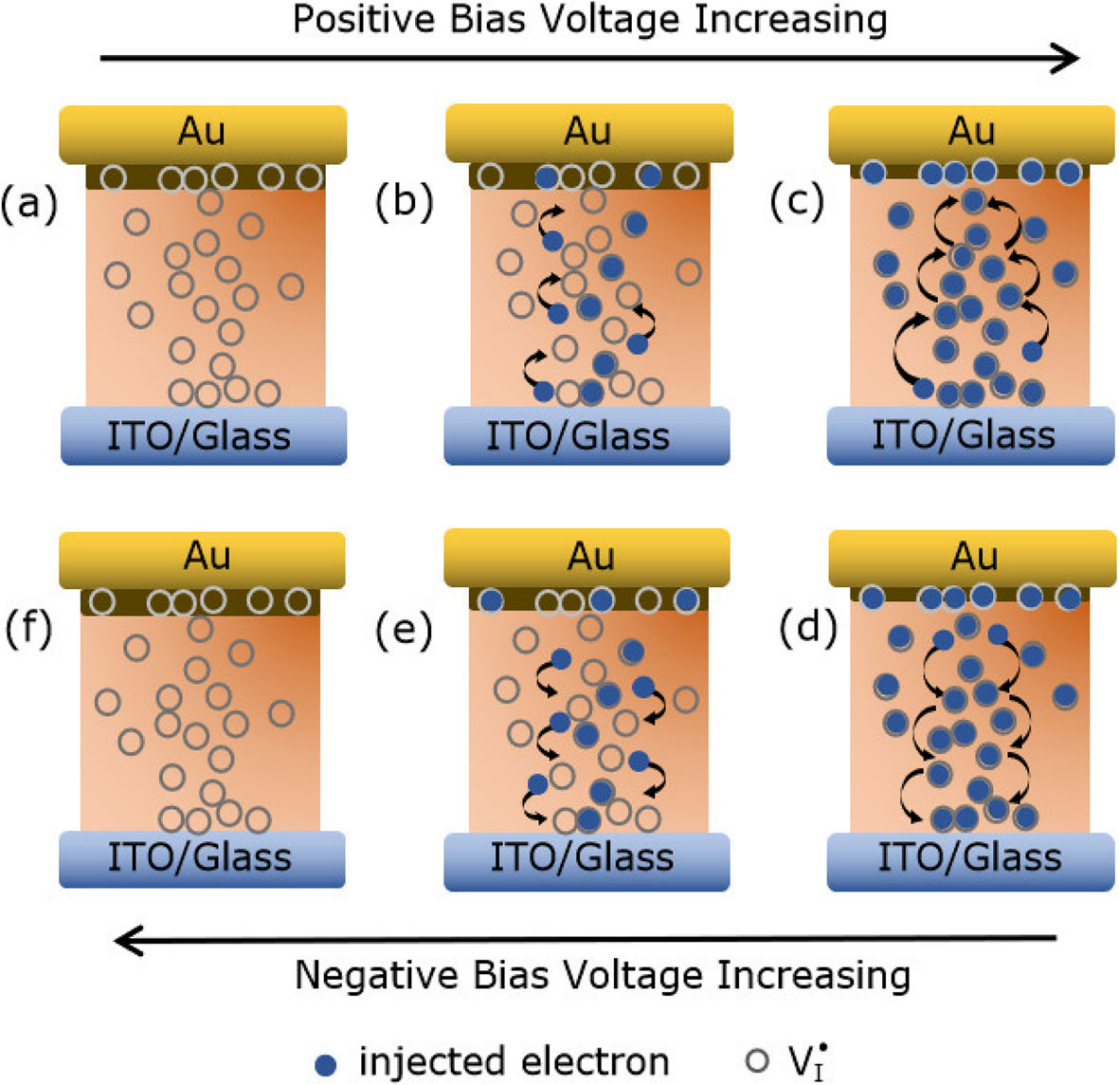
**a**–**f** The schematic of the RS mechanism model in the Au/KCl-MAPIC/ITO/glass cell

As shown in Fig. 7a, in a low positive bias region (0 <*V*<0.2 V), the concentration of thermally generated free carriers is higher than injected carriers in the KCl-MAPIC layer, so the *I*−*V* relationship obeys Ohm’s law:

1 $$ j = qn\mu\frac{V}{d}  $$

where *j* is the transport current density, *q* is the electric charge, *n* is the density of the free electrons in thermal equilibrium, *μ* is the carrier mobility, *V* is the applied voltage, and *d* is the media layer thickness. As the forward voltage increases (0.2 V <*V*<*V*_TFL_), the electrons injected from the bottom ITO electrode are captured by $\mathrm {V}_{\dot {\mathrm {I}}}$ in the KCl-MAPIC layer [Fig. 7b]. The *I*−*V* relationship follows the functional form:

2 $$ j = \frac{9}{8}\theta\varepsilon_{0}\varepsilon_{r}\mu\frac{V^{2}}{d^{3}}  $$

where *θ* is the fraction of free carriers, *ε*_0_ is the permittivity of the free space, and *ε*_*r*_ is the dielectric constant of the insulator. This conductive behavior obeys the SCL conduction mechanism, which is controlled by single shallow traps located closely at the conduction band [9]. When the forward voltage increases to *V*_TFL_, the electrons trapped are activated and released from traps, whereas additional injected electrons immediately fill in these traps. Thus, the traps are always filled up; the conductive behavior switches to the trap-free SCL conduction. The current exponentially increases with the positive bias increasing. The aforementioned process is known as the trapping process. When the forward voltage reaches *V*_SET_, the Au/KCl-MAPIC/ITO cell finally reaches the LRS [Fig. 7c]. The charge traps are filled over time, and the electrons can then hop from trap-to-trap. As the positive bias voltage decreases, the device remains in the LRS due to the high electron concentration in the KCl-MAPIC layer. As illustrated in Fig. 7d, the device still stays in the LRS even though the bias voltage sweeps reversely. Because the trapped electrons cannot be released from the $\mathrm {V}_{\dot {\mathrm {I}}}$ immediately; the carrier concentration remains at a high level. As the negative voltage reaches and crosses *V*_RESET_, the device switches from the LRS to the HRS. The trapped electrons are drawn out from the traps; the electron concentration decreases [Fig. 7e]. When the reverse bias decreases to $V^{*}_{\text {TFL}}$, the current behavior recovers the SCL conduction controlled by single shallow traps. The aforementioned process is known as the detrapping process. As the negative voltage further decreases, the electrons cannot be captured by the traps; the concentration of injected electron concentration is lower than the equilibrium concentration. Therefore, the KCl-MAPIC layer returns to the unoccupied trap state; current behavior transits from SCL conduction to Ohmic conduction [Fig. 7f].

**Fig. 8:**
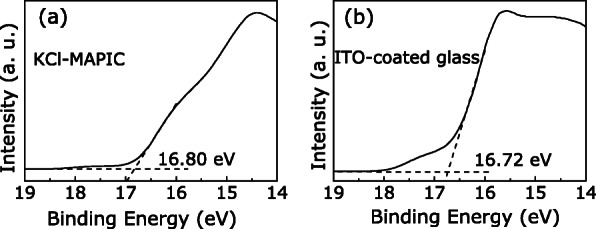
Cut-off regions of **a** the KCl-MAPIC film and **b** the ITO-coated glass

Furthermore, according to reports on the transition process of the current under a bias sweep, we surmise that the bias-induced modification of the barrier height and/or width in the Au/KCl-MAPIC/ITO sandwiches also contributed to the resistive switching [22, 35, 36]. UPS was conducted to confirm the conjecture and examine the contact types of the electrodes/perovskite layer. Figure 8a, b shows the cut-off regions of the KCl-MAPIC film and ITO-coated glass, respectively. The work functions of the film and substrate are calculated as 4.42 eV and 4.50 eV, respectively. These values are similar to results obtained in previous reports [22, 36, 37]. Thus, we confirm that a contact between KCl-MAPIC layer and ITO-coated glass is Ohmic due to their similar work functions. However, it is well known that the work function of Au is about 5.0 eV [22, 35]. This value is larger than that of the KCl-MAPIC film. Therefore, a barrier forms at Au/KCl-MAPIC interface. As shown in Fig. 7b, electrons start to drift towards the Au electrode and be captured by the $\mathrm {V}_{\dot {\mathrm {I}}}$ depletion layer near the Au/KCl-MAPIC interface under a positive voltage. When the forward voltage reaches *V*_SET_, the holes are full-filled, lead to the Schottky-like barrier lowing, and the depletion layer thinning [Fig. 7c]. The contact between KCl-doped MAPIC layer and Au electrode becomes a quasi-ohmic contact, and the device switches from the HRS to the LRS. As shown in Fig. 7d–f, when the bias sweeps in reverse direction and increases to *V*_RESET_, the trapped electrons are pulled out from hole traps and the barrier recovers to the original stat; the electrons injected from the Au electrode are obstructed. Thus, the carrier concentration decreases in the KCl-MAPIC layer; the Au/KClMAPIC/ITO device switches from the LRS to the HRS.

## Conclusions

High-quality KCl-doped MAPIC films were prepared using the low-temperature one-step solution synthesis. The appropriate dosage of potassium chloride doping could help MAPIC films grow to the good quality with high coverage and dense surface. The memory cells consisting of Au/KCl-MAPIC/ITO/glass exhibited a tri-state RS behavior after applying different *V*_SETs_ at room temperature. Cycling endurance (>140 cycles) and data retention (≥1000 s) demonstrated that the Au/KCl-MAPIC/ITO/glass devices have the potential for multilevel storage in ReRAM. The analysis of conductive processes revealed the $\mathrm {V}_{\dot {\mathrm {I}}}$ traps-controlled SCLC mechanism contributed to the RS behavior. Furthermore, the modulation of the Au/KCl-MAPIC barrier under the applied bias was also responsible for the resistive state switching in the carrier injection-trapping/detrapping process.

## Supplementary information

**Additional file 1** Supporting Information.

## Data Availability

All data generated and analyzed during this study are included in this article and the attached supporting information.

## Abbreviations

ReRAM: Resistive switching random access memory
OHPs: Organometal halide perovskites
MAPIC: CH_3_NH_3_PbI _3−*x*_Cl_*x*_
KCl: Potassium chloride
ITO: Indium tin oxide
RS: Resistive switching
*I*−*V*: Current-voltage
LRS: Low resistance states
HRS: High resistance state
*V*_SET_: Set voltage
*V*_RESET_: Reset voltage
*V*_r_: Read voltage
$\mathrm {V}_{\dot {\mathrm {I}}}$: Iodine vacancy
SCLC: Space-charge-limited current
*V*_TFL_: Trap-filled limit voltage
XRD: X-ray diffractometry
XPS: X-ray photoelectron spectroscopy
UPS: Ultraviolet photoelectron spectroscopy
SEM: Scanning electron microscopy
