# Supplementary material for: Multilevel Resistive Switching Memory Based on a CH3NH3PbI 3−xClx Film with Potassium Chloride Additives
Source: Nanoscale Res Lett. 2020 Jun 5;15:126. doi: 10.1186/s11671-020-03356-3 (PMC7275113; doi:10.1186/s11671-020-03356-3)
Supplement: Supplementary file 1 — Additional file 1 Supporting Information. [file 11671_2020_3356_MOESM1_ESM.docx]

**Supporting Information**

**Multilevel resistive switching memory based on a
CH_3_NH_3_PbI_3-_*_x_*Cl*_x_* film with potassium chloride
additives**

Fengzhen Lv^*^, Kang Ling^†^, Tingting Zhong, Fuchi Liu^*^, Xiaoguang Liang, Changming Zhu, Jun Liu and Wenjie Kong

*College of Physics and Technology, Guangxi Normal University, Yucai Road, Guilin 541000, China*

^*^Corresponding author: E-mail:

[lvfzh17@mailbox.gxnu.edu.cn,](mailto:lvfzh17@mailbox.gxnu.edu.cn,) liufuchi@gxnu.edu.cn


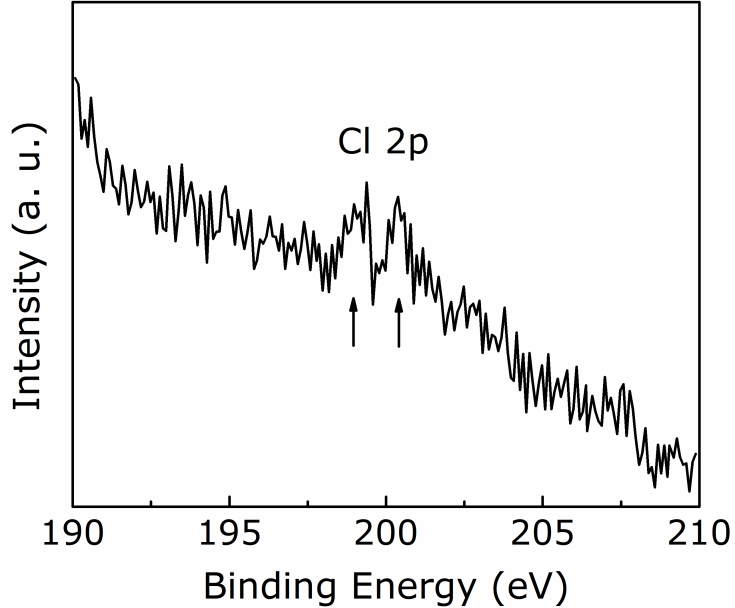


**Fig. S1** Characteristic peak binding energy of Cl 2*p*_3/2_ and Cl 2*p*_1/2_ (199.0 eV and 200.4 eV) highlighted by the arrows.


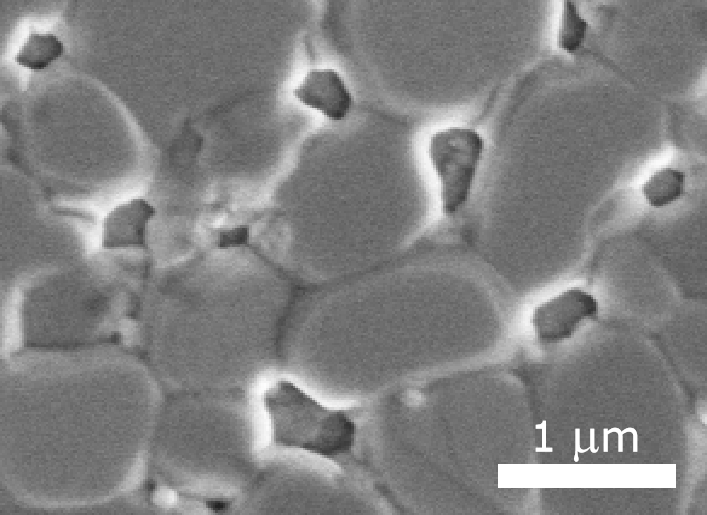


**Fig. S2** The top-view SEM image of the MAPIC films without the KCl additive.





**Fig. S3** (a) The *I*-*V* characteristic of Au/MAPIC/ITO/Glass under the sweeping voltage of 0 V→1 V→0 V→-1 V→0 V. (b) The semi-logarithmic plots of the *I-V* curve of the Au/MAPIC/ITO/Glass devices under different *V*_SETs_ (0.8 V and 1 V). The numbers and arrows represent the voltage sweeping sequences and directions, respectively.
